# Evaluation of non-enrolment of children in all English state schools: linked, administrative data cohort study of children receiving social care and their peers.

**DOI:** 10.23889/ijpds.v7i3.1775

**Published:** 2022-08-25

**Authors:** Matthew Jay, Louise Mc Grath-Lone, Bianca De Stavola, Ruth Gilbert

**Affiliations:** 1 UCL

## Objectives

All children have a right to education, but research indicates that those receiving children’s social care (CSC) services are at increased risk of non-enrolment in school, including through off-rolling (illegal exclusion). We aimed to use administrative data to estimate the association between CSC history and non-enrolment in secondary school.

## Approach

Using the National Pupil Database (data on all English state school enrolments), we identified a cohort of 1,059,781 pupils aged 11 in 2011 and 2012. Children were categorised as having a history of being children in need (CiN), on child protection plans (CPPs) or looked after (CLA) using linked data from CSC services. We estimated the proportion of children not enrolled across ages 12 to 16 by CSC history. We then assessed with regression modelling the association between CSC history and non-enrolment in years 10/11. We also examined variation in overall non-enrolment rates between local authorities and regions.

## Results

Of children without CSC history, 3.8% had 1 or more non-enrolments across ages 12 to 16. This proportion was higher among children with a history of being CiN (8.1%), on a CPP (9.4%) or being CLA (10.4%). The odds of non-enrolment in years 10/11 were higher among those with CLA history vs non-exposed peers (OR 4.76, 95% CI 4.49-5.05) as well as in those with CPP (3.60, 3.39-3.81) and CiN history (2.53, 2.49-2.58). History of special educational needs and disability (SEND) further increased non-enrolment odds. These associations and interactions persisted after adjusting for confounders. Non-enrolment rates were highest in the London region and varied significantly between local authorities.

## Conclusion

Our findings show that children with CSC history (especially those with SEND) are more likely to be non-enrolled in secondary school than other children. Work is needed to understand the non-enrolment mechanisms, which may include illegal off-rolling and other exclusionary practices, to improve the education of children with CSC history.

